# Real-World Insights into the Impact of Durvalumab on Stage III Unresectable Non-Small Cell Lung Cancer—A Narrative Review

**DOI:** 10.3390/cancers17050874

**Published:** 2025-03-03

**Authors:** Giorgio Facheris, Gianluca Cossali, Jessica Imbrescia, Salvatore La Mattina, Eneida Mataj, Nicole Meli, Giulia Volpi, Luca Triggiani, Andrea Emanuele Guerini, Guido Levi, Salvatore Grisanti, Michela Buglione di Monale e Bastia, Paolo Borghetti

**Affiliations:** 1 Radiation Oncology Department, ASST Spedali Civili and University of Brescia, 25123 Brescia, Italy; g.cossali@unibs.it (G.C.); e.mataj@unibs.it (E.M.); luca.triggiani@unibs.it (L.T.); a.e.guerini@gmail.com (A.E.G.); michela.buglione@unibs.it (M.B.d.M.e.B.); paolobor82@yahoo.it (P.B.); 2 Radiation Oncology Unit, Department of Oncology and Hematology, University Hospital of Modena, 41124 Modena, Italy; imbresciajessica@gmail.com; 3 Departement of Radiation Oncology, San Matteo Hospital Foundation Istituto di Ricovero e Cura a Carattere Scientifico (IRCCS), 27100 Pavia, Italy; salvatore.lamattina.1@gmail.com; 4 Oncology, Department of Medical and Surgical Specialties, Radiological Sciences, and Public Health Medical, ASST-Spedali Civili, University of Brescia, 25123 Brescia, Italy; nicolemeli1994@gmail.com (N.M.); grisanti.salvatore@gmail.com (S.G.); 5 Azienda Ospedaliera Universitaria Integrata Verona, Radiation Oncology, 37126 Verona, Italy; g.volpi50@gmail.com; 6 Pulmonology Department, ASST Spedali Civili di Brescia, 25123 Brescia, Italy; guido.levi@yahoo.it; 7 Department of Clinical and Experimental Sciences, University of Brescia, 25121 Brescia, Italy

**Keywords:** lung cancer, pacific, real world, durvalumab, NSCLC, radiotherapy, chemotherapy, immunotherapy, narrative review

## Abstract

Introduction and Aim: Stage III Non-Small Cell Lung Cancer (NSCLC) has a poor prognosis, with median survival ranging from 9 to 34 months. The PACIFIC trial demonstrated that durvalumab after platinum-based chemoradiotherapy (CRT) improves overall survival (OS) and progression-free survival (PFS). This review evaluates real-world evidence (RWE) on durvalumab’s efficacy and safety, focusing on patient characteristics, prognostic factors, treatment protocols, and outcomes beyond progression. Materials and Methods: A literature search of PubMed, Embase, and Google Scholar identified 49 observational studies published from January 2017 to August 2024 on unresectable stage III NSCLC. Clinical trials, early-stage disease, and alternative treatments were excluded. Results: Compared to the PACIFIC trial, real-world patients were older, had poorer ECOG performance (≥2), and more comorbidities like COPD. Despite this, durvalumab provided consistent survival benefits. Positive prognostic factors included non-squamous histology, high PD-L1 expression, and timely durvalumab initiation (≤42 days post-CRT). Most radiotherapy regimens mirrored PACIFIC (54–66 Gy). Concomitant CRT was used in 90% of cases, with sequential CRT for frail patients. Chemotherapy regimens varied. Immune-mediated pneumonitis was a major adverse event, with incidence rates between 15% and 100%. Severe cases led to treatment discontinuation, impacting survival. Treatment beyond progression remains uncertain, with limited benefits from immunotherapy rechallenge. Conclusions: RWE supports durvalumab’s efficacy, emphasizing the need for personalized treatment strategies and further research to improve long-term outcomes.

## Simple Summary

Stage III Non-Small Cell Lung Cancer (NSCLC) has a poor prognosis, but the PACIFIC trial showed that durvalumab after chemoradiotherapy (CRT) improves survival. This review examines real-world evidence (RWE) on durvalumab’s effectiveness and safety. Studies have shown that real-world patients are often older and have more comorbidities than those in clinical trials, yet they still benefit from durvalumab. Key prognostic factors include non-squamous histology, high PD-L1 expression, and timely treatment initiation. Radiotherapy and chemotherapy protocols vary slightly, with some patients receiving sequential CRT. Immune-mediated pneumonitis is a significant side effect, sometimes leading to treatment discontinuation. The role of treatment beyond progression remains unclear, with limited benefits from re-challenging immunotherapy. Overall, real-world data support durvalumab’s efficacy, highlighting the need for personalized treatment approaches and further research to optimize patient outcomes.

## 1. Introduction

Lung cancer is a leading cause of cancer incidence and mortality worldwide, accounting for 18.4% of total cancer deaths [1]. Non-small cell lung cancer (NSCLC) accounts for more than 80% of all lung cancer cases [2]. As reported by the Surveillance, Epidemiology and End Results (SEER) registry, the incidence of NSCLC is 42.6 per 100,000 population [3]. Stage III NSCLC represents a heterogeneous disease both in terms of extension of the primary tumor and involvement of the mediastinal nodal stations. A wide debate is ongoing within the oncological community concerning the resectability of stage III NSCLC due to an improvement of the surgical skills and the advent of immunotherapy and targeted therapies in the perioperative treatment [4]. Nevertheless, an approach with chemo-radiotherapy (CRT) remains the standard of cure for all cases that cannot receive a radical surgery or for those patients who are not suitable for surgery due to age, comorbidities, or clinical conditions that roughly represent 25% of all NSCLC at presentation. For these unresectable NSCLC patients, the addition of the anti-PD-L1 immunotherapy with durvalumab after platinum-based CRT has been recognized as the new standard of care according to the results of the phase III PACIFIC trial [2]. Both overall survival (OS) and progression-free survival (PFS) improved with the use of durvalumab after concurrent CRT, without unacceptable toxicity and/or deterioration of patients’ quality of life [3].

While long-term survival benefit with the addition of durvalumab was confirmed in subsequent updates of the PACIFIC trial [4], an increasing need for the translation of these data into daily clinical practice emerged in the years following initial publication of the study. Indeed, real-world (RW) data not only evaluate whether a benefit can be equally transferred from a selected to an unselected patient’s population, but they can also explore controversial and debated aspects that emerge from clinical practice right after the occurrence of practice-changing studies, as well as investigate the role of a standard treatment within an ever-changing world [5,6].

This prompted the design of the PACIFIC-R trial that involved patients coming from the expanded access program (EAP) of durvalumab [5].

In this narrative review, we aimed to critically analyze information regarding unmet needs and critical issues, like the differences in patient characteristics, prognostic factors, and CRT regimen used in RW studies or the incidence of immune-related pneumonitis, PD-L1 status, and oncogenic driver mutation in a non-study-selected population, and finally, treatment beyond progression after the PACIFIC regimen. Data from studies reporting patients’ characteristics and prognostic factors, CRT protocols and regimens, immune-related pneumonitis, PD-L1 status and oncogenic driver mutation, and the treatment after progression for patients treated with the PACIFIC regimen were collected and reviewed. The key point of our analysis is to highlight that, although patients selected for clinical trials are usually different in terms of age, comorbidities, or performance status from those encountered in real-world clinical practice, the data related to the analyzed points show a certain degree of adherence to those from the registrational PACIFIC study, thus validating its value even in contexts different from the more controlled settings of clinical trials.

## 2. Materials and Methods

A literature review was performed accessing the online databases PubMed, Embase, and Google Scholar to identify English-language real-world (RW) studies on the clinical application of durvalumab in NSCLC. The search covered the period from 1 January 2017 to 31 December 2024. The following keywords were searched independently by two reviewers: “lung cancer”, “lung carcinoma”, “NSCLC”, and “durvalumab”. The search string used was {[(lung cancer) OR (lung carcinoma)] OR (NSCLC)} AND (durvalumab). Any differences in the selection process were resolved by consulting between the two authors. The bibliography of the included studies and previous reviews was hand-searched to check for any missed relevant studies.

A total of 392 records was retrieved, and a preliminary selection was conducted based on the titles and abstracts of the articles. Duplicate entries were excluded, along with manuscripts not published in English, and abstracts or posters lacking full-text availability.

The remaining studies were then extracted and checked for eligibility. The eligibility criteria were defined as: (1) Observational studies reporting the use of consolidation durvalumab for unresectable stage III NSCLC after curative CRT. (2) Studies reporting data on OS, PFS, or adverse events. We excluded studies on early-stage NSCLC, combining CRT with surgery, using palliative therapy, tyrosine kinase inhibitors, or other ICIs instead of durvalumab. Studies with duplicate data, clinical trials, review articles, and case reports were also excluded.

A total of 51 studies meeting the criteria was identified and included in the review. The analysis was then conducted analyzing each article about the following research topics: patient characteristics and prognostic factors, CRT regimen, immune-related pneumonitis, PD-L1 status and oncogenic driver mutation, and treatment beyond progression after the PACIFIC regimen. As a narrative review, the goal of this work is to identify and summarize what has previously been published with the main aim of critically analyzing information regarding unmet needs and critical issues of this specific disease setting.

**Figure 1:**
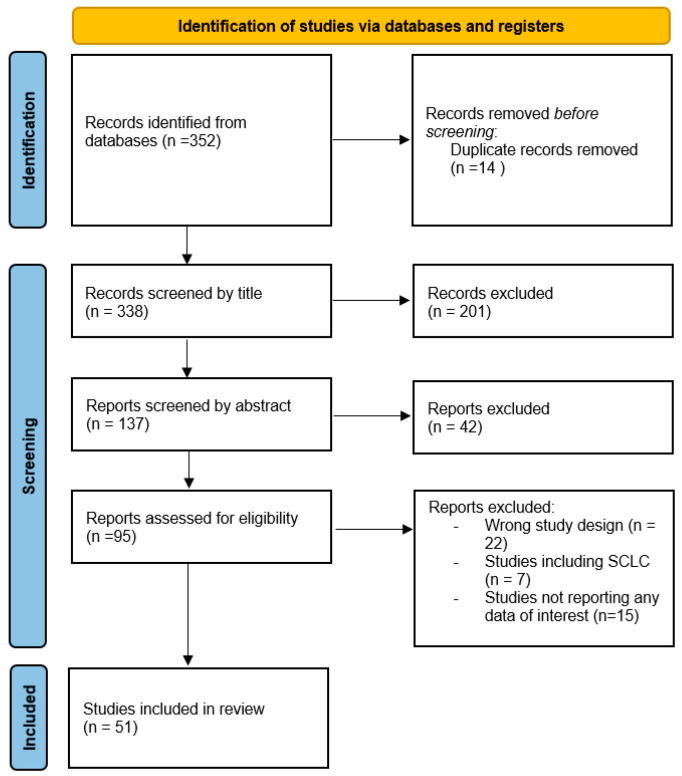
PRISMA diagram of the screening and selection flow.

The PRISMA diagram below illustrates the screening and selection flow (Figure 1).

## 3. Results and Discussion

### 3.1. Patient Characteristics

The demographic and clinical characteristics of patients as extracted by the retrospective real-world analyzed studies are summarized in Table 1.

Notably, the median age of patients, in over 70% of the studies, was higher than in the PACIFIC trial. In the studies by Waterhouse et al. [8] and Takeda et al. [19], the median age of the 528 and 107 patients was as high as 70 years. Regarding gender, the PACIFIC study reported 70.2% males; the retrospective RW data confirmed a male predominance, although in some cases, this was less pronounced: only Waterhouse et al. [8] Preti et al. [10] reported the equivalence between male and female with percentages of male gender of 51.5% [12], 51.7%, and [14] 50.9%, respectively. Only Trinh [23] reported a female prevalence.

Data on race, reported in 5/18 papers, showed Caucasians as a much more frequent race (78% in Moore’s study [7], 70.6% in Waterhouse’s study [8], 74.1% in Sankar’s study [11]).

While the PACIFIC trial enrolled patients having an ECOG performance status of 1 or less, RW studies have shown an open approach to patients more commonly encountered in real life. Except for a few studies where performance status data were missing, many studies described the presence of patients with ECOG > or equal to 2, up to 16% [7].

Regarding comorbidities, some studies registered a median Charlson Comorbidity Index (CCI) score of 6 [7]; a CCI higher or equal to 6 was reported in 51% of patients [11]. Notably, patients with COPD were sometimes described as comorbidity of particular interest for incidence: Moore et al. [7] reported a COPD rate of 70%, and Borghetti et al. [14] documented 57.1% of patients with COPD, of whom 13.5% had grade 3 or greater. 

The proportion of current smokers in RW studies averaged 35.7%, significantly higher than the 16.6% in the PACIFIC trial. 

The most common histotype in the PACIFIC trial was adenocarcinoma (57%). The data from the studies included in this review were generally in line with PACIFIC results, with the exception of Moore et al. [7], who reported 50% squamous cell carcinoma (SCC) and 43% non-squamous; Park et al. [18], who reported 52.2% SCC and 40.8% non-squamous; and Takeda et al. [19], who reported 50% SCC and 43.8% non-squamous.

The median overall survival for the studies reporting these data was 38.3 months (ranging from 18.2 and 58.7 months).

Finally, RW patients tended to be older, with a higher proportion of comorbidities, such as COPD and cardiovascular diseases, and a larger percentage of smokers compared to the PACIFIC cohort. These factors suggest that real-life patients are often frailer and may not fit the strict inclusion criteria of clinical trials. Despite these differences, the integration of durvalumab with CRT remained a viable treatment option for such patients, offering promising results even in those with higher comorbidity scores or lower performance status. This underscores the need for clinicians to consider these variabilities when translating trial results into everyday practice, as it is evident that the PACIFIC regimen remains a robust standard of care for patients with unresectable stage III NSCLC, including more fragile populations.

### 3.2. Prognostic Factors

The PACIFIC trial identified several positive prognostic factors for survival. Univariate analysis highlighted younger age (<65 years), objective tumor response during prior CRT (vs. stable disease), non-squamous histology (vs. squamous), ECOG-PS 0 (vs. PS 1), cisplatin-based chemotherapy (vs. carboplatin), and Asian race (vs. White) as favorable factors for OS. Non-squamous histology and Asian race were also prognostic for better PFS. Multivariable analyses confirmed these factors, adding female gender for OS and stage IIIA (vs. IIIB) for PFS.

Real-world (RW) studies further explored these factors, identifying PD-L1 expression and EGFR status as relevant prognostic markers. In PACIFIC-R, a trend of better PFS was observed in patients with PD-L1 ≥1%, stage IIIA, non-squamous histology, concomitant CRT, cisplatin-based chemotherapy, start of durvalumab ≤42 days after RT, and wild-type EGFR, although differences were not statistically significant [8]. PACIFIC-KR, the first multicenter RW study in South Korea, found stage IIIA (vs. IIIB–C), N0-1 (vs. N2–3), and PD-L1 TPS >25% and >50% (vs. <25% and <50%) as favorable factors for PFS. The limited number of PD-L1-negative cases (19 out of 157) affected the analysis [18].

PD-L1 >1% was confirmed as a predictor of OS benefit from durvalumab, while tumor PD-L1 expression was supported as a prognostic factor for OS and PFS in stage III NSCLC patients [24]. However, studies indicated that EGFR status did not modify durvalumab’s effect on PFS, with no significant differences in median PFS between patients with and without driver genomic alterations (BRAF/KRAS mutations, ALK/ROS1 rearrangements) [9,25].

The Zhang meta-analysis suggested that only performance status significantly influenced PFS, while age, time to durvalumab initiation, and PD-L1 status significantly impacted pneumonitis rates but not survival outcomes [24]. Although elderly patients had similar survival outcomes to younger ones, they often took longer to recover from CRT toxicity, delaying immunotherapy initiation and reducing pneumonia risk. Strong negative prognostic factors such as ECOG PS 2–3 and tumor progression during induction chemotherapy or CRT were confirmed [6].

A retrospective analysis showed that only stage IIIA (vs. IIIB/C) was associated with improved OS, but not PFS, with no association between OS/PFS and performance status, immune disease history, PD-L1 receptor status, delayed durvalumab start (>42 days), or adverse event development [13]. In the US community setting, it was found that OS/PFS were shorter in patients who discontinued durvalumab early due to adverse events, with older patients and males at higher risk of death or progression [8].

While RW data often come from retrospective cohorts with selection biases, they provide insights into real-world adherence to RCT outcomes. Overall, these findings support the broad applicability of PACIFIC trial data in clinical practice, emphasizing PD-L1 expression as a key prognostic marker, while the role of EGFR status remains uncertain.

### 3.3. Chemo-Radiotherapy Regimen

The globally recognized standard of care for patients with unresectable stage III non-small cell lung cancer (NSCLC) is normo-fractionated radiotherapy, with a total dose of 60 Gy delivered 30 daily fractions of 2 Gy per fractions, concomitantly platinum-based doublet chemotherapy, followed by maintenance immunotherapy with durvalumab.

This treatment protocol represents the sum of several milestones. In 2010, Auperin et al. [26] published a meta-analysis demonstrating survival benefits in the concomitant group. Subsequently, in 2015, the RTOG 0617 showed that dose escalation to 74 Gy was not superior to 60 Gy and potentially harmful [27]. Finally, in 2017, Antonia et al. demonstrated that the addition of durvalumab improved overall survival rates [2].

So, it is intriguing to examine within the selected studies how closely real-life practices align with or differ from this standard. The detailed descriptions of all selected articles are provided in the table.

In this analysis, approximately 31,700 patients have been included. CRT was administered concomitantly in approximately 90% of cases. Sequential treatment was reserved for only about 10% of cases, and the reasons for choosing sequential treatment were rarely declared, but could be inferred from clinical practice, suggesting that sequential combination was reserved for more fragile, older, or with multiple comorbidities patients, in which concomitant treatment might lead to intolerable toxicity.

The most widely used chemotherapy doublet was carboplatin in combination with paclitaxel. Furthermore, vinorelbine played a significant role in combination to platinum derivatives. A weekly schedule appeared to be the most common regimen, but probably there was a significant bias related to the limited availability of data about the chemotherapy schedule.

Regarding treatment data of radiotherapy, the significant point to note was that most selected studies did not provide technical details. Often, only the median, minimum, and maximum delivered doses were reported without further details on fractionation or techniques.

Nevertheless, it can be declared that in about 90% of cases, the delivered dose ranged between 54 and 66 Gy, according to the doses allowed in PACIFIC trials (60 Gy +/− 10%). Reported doses below 54 Gy were rare, while cases with doses exceeding 66 Gy were more frequent. Only two studies evaluated dose escalation up to 74 Gy in sequential RCT, suggesting that sequential high-dose chemoradiotherapy followed by durvalumab might be similar to the standard of care, but larger prospective studies are needed to confirm this hypothesis [28,29].

The normo-fractionated treatment at 2 Gy/fraction was undoubtedly the most used. The use of hypofractionation in 20–25 fractions was limited; likewise, it had never been specified for which patients it was reserved. It could be assumed that hypofractionation was proposed to patients with reduced performance status in order to reduce the total treatment time.

Compared to 3D techniques, the use of modern radiotherapy techniques such as Intensity-Modulated Radiation Therapy (IMRT) or Volumetric Modulated Arc Therapy (VMAT) was more represented.

However, there is a notable lack of declared data on the use of Image-Guided Radiation Therapy (IGRT), despite its essential role in ensuring treatment accuracy and reducing toxicity. This suggests that IGRT might be widely used but underreported, as authors may assume its routine application. The absence of standardized reporting on radiotherapy details introduces variability in treatment approaches, making direct comparisons challenging.

Finally, despite the administered doses, the timing of RCT, and the drugs adopted, the data about 1-, 2-, and 3-year OS were nearly overlapping in all cases and corresponded approximately to those reported in the PACIFIC study, except for the 3-year OS, which was higher (presumably due to the reduced percentage of studies reaching 3-year OS data). This consistency in OS outcomes raises an intriguing question: why do different treatment approaches lead to similar survival rates? Potential explanations include the inherent robustness of CRT as a backbone for stage III NSCLC treatment, the dominant role of durvalumab in driving survival benefits, and the possibility that differences in radiotherapy dose and fractionation may have a limited impact within the PACIFIC-defined range (54–66 Gy). However, the lack of granular data prevents definitive conclusions, highlighting the need for more detailed prospective studies evaluating the interplay between radiotherapy techniques and long-term outcomes.

Table 2 provides OS data for arms where durvalumab was administered.

### 3.4. Immune-Related Pneumonitis

Pneumonitis is a serious and potentially life-threatening adverse event in patients receiving CRT and durvalumab as maintenance for unresectable locally advanced NSCLC. This review aimed to provide an overview of the existing data on pneumonitis in these patients, focusing on its incidence, risk factors, and clinical presentation. Comparison to observational RW data was limited by the heterogeneity of reporting adverse events in trials.

Pneumonitis was commonly diagnosed on the base of the presence of symptoms, timing of RT or durvalumab, radiographic changes such as the development of ground glass opacities, and the exclusion of alternative causes. Among the studies, pneumonitis was recorded according to Common Terminology Criteria for Adverse Events (CTCAE) version 4.0 and version 5.0 [54]. 

All the identified studies included at least one arm treated with combined therapy. In the studies reporting pneumonitis data, the median age was 67 years (range 29–90 years). The proportion of patients with an ECOG performance status of grade 0–1 ranged from 61% to 100% (mean 92.1%). The mean of patients with Chronic Obstructive Pulmonary Disease of any stage was 46.9%. There were poor results on the pulmonary function tests performed for patients before treatment.

Most applied radiation therapy techniques were IMRT and VMAT, but also 3D-CRT and Proton beam therapy (PBT). In the latter case, the technique was chosen when the doses to normal tissue were likely to exceed acceptable constraints. However, the technique was not reported in all studies. The most prescribed dose was 60 Gy in 30 fractions, and the median RT dose was 60 Gy. Dosimetric parameters were reported in 8 studies. The median lung V20 was 20.5% (range 16%–27%). The median MLD was 14.1 Gy (range 12–16.7).

Following CRT, patients received an average of 14 (range 1–47) doses of durvalumab. The mean time elapsed between finishing CRT and beginning durvalumab was 33.75 (range 0–238) days in all patients. 

The cumulative incidence of any grade pneumonitis in patients undergoing combined treatment with CRT and durvalumab varies across clinical studies but was generally reported to be between 15.6% [48] and 100% [37], with a mean of 46.84%. An incidence of 100% has been reported in papers where small number of patients were included in the study (12 patients) and where the 50% of the events were grade 1 pneumonitis. Considering only the grade 2 or higher pneumonitis events, the mean incidence was 36.05% (range 15.6%–58.5%), while the mean incidence of grade 3 or higher events was 6.75% (range 0%–16.9%).

Most studies do not specifically distinguish Radiation-Induced Lung Injury (RILI) cases from immunotherapy-related pneumonitis when reporting adverse reactions, due to the clinical difficulty to distinguish the exact point. The PACIFIC trial did not describe these two categories separately [2].

Diamond et al. [49] found a higher rate of RILI than immunotherapy-related pneumonitis (24% vs. 6.5%, respectively), while 1.6% of cases were equivocal. Moreover, they found that the number of cycles of durvalumab was correlated with development of pneumonitis. Park et al. [48] also confirmed a higher incidence of RILI (36,3%) than that of irAE (14.6%). In contrast, in a retrospective cohort of 1005 patients treated with combined therapy [34] attribution to immunotherapy was higher (9% vs. 5.57% in pts treated with CRT alone). In the same study, it was found that the use of durvalumab was associated with higher risk of grade 2 pneumonitis, but it did not increase grade ≥ 3 pneumonitis incidence.

The mean of patients who had durvalumab consolidation therapy interrupted due to suspected pneumonitis was 26.35% (range 3.85–53.7). Saad et al. [13] found that the interruption of durvalumab because of an AE was associated with decreased of both OS and PFS, therefore suggesting caution when considering discontinuation of adjuvant durvalumab.

Several risk factors related to the patient, tumor, and treatment could influence the probability of developing pneumonitis during combined treatment regimens. 

For all patients, the already-known risk factors, such as history of smoking, COPD, interstitial lung disease, V20, V30, and MLD, were associated with increased risk of RILI. A meta-analysis from Wang et al. [34] based on the efficacy and safety of consolidation durvalumab identified older age (patients >65 years old) and Asian ethnicity as risk factors for pneumonitis. Adding PD-1/PD-L1 inhibitors increased the risk of G1–G2 pneumonitis but did not increase grade ≥ 3 pneumonitis incidence [55]. Moreover, it appears that the time interval of durvalumab administration less than 42 days did not affect the risk of pneumonitis [51].

The variability in pneumonitis incidence across RW data can be attributed to several factors, including differences in patient populations, treatment protocols, and diagnostic approaches. First, the presence of comorbidities such as COPD or interstitial lung disease significantly increases the likelihood of pneumonitis, which may not be consistently documented in all RW studies. The heterogeneity in patient health status, including age and performance status, further complicates the comparison of outcomes across studies. Additionally, variations in radiation therapy techniques and dosimetric parameters (e.g., lung V20, MLD) can also contribute to differences in pneumonitis incidence. Furthermore, the lack of uniformity in the timing of durvalumab initiation after CRT completion and the number of treatment cycles could explain some of the observed variability in pneumonitis development and severity.

In RW settings, the management of pneumonitis may vary significantly depending on the treating institution’s experience, the available resources for early detection, and the clinical threshold for interrupting or discontinuing durvalumab. Because pneumonitis symptoms overlap with other pulmonary conditions, especially in patients with pre-existing lung diseases, accurate diagnosis remains challenging, further complicating clinical management. Additionally, RW studies often lack standardized protocols for reporting adverse events, leading to inconsistent definitions of pneumonitis severity and underreporting in some cases. Given the potential for pneumonitis to impact both OS and PFS, the decision to withhold durvalumab therapy requires careful consideration of the trade-off between potential harm and therapeutic benefit, highlighting the need for individualized patient management strategies [56].

Available data for pneumonitis in the analyzed articles are summarized in Table 3.

### 3.5. PD-L1 and Driver Mutations

Advances in cell biology have identified key genomic alterations (dGA) in NSCLC, including mutations in EGFR, KRAS, BRAF, and rearrangements in ALK, ROS1, NTRK, and RET. Immune-checkpoint inhibitors (ICIs) are central to tumor immunotherapy, with PD-L1 expression as the only approved biomarker for predicting ICIs’ outcomes in NSCLC. The PACIFIC trial showed OS and PFS benefits in patients with PD-L1 ≥1%, leading the EMA to approve durvalumab for these patients, while the FDA approved it regardless of PD-L1 expression. Real-world data could clarify if clinical trial results translate to daily practice. The RW research by Preti et al. [10] revealed that OS did not depend on PD-L1 status; however, this analysis had different limitations, such as the small sample size (N = 118) and the heterogenous population of the patients enrolled (both stage III and oligometastatic patients).

Bryant et al. [25] found better PFS in NSCLC patients with PD-L1 >50% compared to those with PD-L1 <1%. No survival difference was observed in PD-L1 <1% patients who did not receive durvalumab, unlike those with higher PD-L1 levels. However, this study lacked PD-L1 data for untreated patients. In contrast, a retrospective analysis [34] of 551 NSCLC patients, including those untreated with durvalumab, found PD-L1 status unrelated to PFS in untreated patients, while PFS differences emerged only in durvalumab-treated patients with PD-L1 ≥1%, supporting PACIFIC trial results.

The PACIFIC-R study [17] confirmed longer PFS in PD-L1 ≥1% patients, though PFS benefits were also seen in PD-L1 <1% patients. A three-year follow-up reinforced this trend. PD-L1 remains the primary biomarker for guiding treatment, but RW studies vary due to differences in study design, PD-L1 stratification, and testing methods. Some have divided patients into PD-L1 <1%, 1–49%, and >50% groups, while others have used a binary classification. Furthermore, PD-L1 data were sometimes unavailable for untreated patients, and variations in testing methods impacted reported results.

Another debated issue is immunotherapy use in patients with PD-L1 >1% and oncogenic driver mutations. Based on PACIFIC data, an ESMO consensus advises against immunotherapy post-cCRT in EGFR/ALK-mutated patients [5]. Limited data exist for other mutations, but poorer PFS was observed in ERBB2/EGFR-mutated unresectable stage III NSCLC patients treated with durvalumab. A retrospective study [25] of 323 stage III NSCLC patients treated with CRT and durvalumab showed different responses across genetic subgroups, with KRAS-mutated patients benefiting the most. A study found that KRAS-mutated NSCLC patients receiving durvalumab after chemoradiation had progression-free survival comparable to those without actionable mutations, though they experienced higher rates of extrathoracic progression. Additionally, an Austrian registry study demonstrated significantly improved 2-year overall and progression-free survival in KRAS-mutated NSCLC patients treated with immunotherapy compared to those who did not receive it. These findings highlight the potential of immunotherapy in this subgroup while emphasizing the need for further research into resistance mechanisms and optimal treatment strategies [4,62,63].

New strategies are needed for oncogene-driven disease, such as EGFR TKIs before cCRT, which may reduce tumor burden [64]. The LAURA trial recently showed positive results for adjuvant osimertinib after cCRT in EGFR-mutated patients [65]. Given the lack of immunotherapy benefits in patients with driver mutations, current clinical trends avoid ICIs in this subgroup. Ongoing trials, including KEYVIBE006 (NCT05298423), SKYSCRAPER-01 (NCT04294810), and PACIFIC-9 (NCT05221840), are now enrolling only wild-type patients, reinforcing the absence of ICIs’ advantages in mutated cases.

More tailored approaches are needed to answer the unmet needs: new tools are being evaluated to personalize the patients’ treatment, such as ctDNA, radiomics, and PET-CT guidance [66]; however, to date, a baseline Next-Generation Sequencing (NGS) for all the new diagnosis should be recommended because it may play a crucial role in the daily clinical decision making. 

### 3.6. Treatment Beyond Progression

Patients with inoperable NSCLC because of locally advanced stage, have a high risk to relapse after radical CRT mainly because of a high local nodal burden of disease. In the 5-year update of the PACIFIC trial, 56.3% of patients in the durvalumab arm experienced disease progression, and the benefit of durvalumab addition was inferior in patients with stage IIIB disease, confirming that higher nodal stage is a major negative prognostic factor for progression and distant metastases [9].

Thus, “what to do after pacific regimen” progression remains a burning question, as there are no prospective data suggesting second-line treatments. In the same 5-year update of the PACIFIC trial, 33% of progressed patients received chemotherapy, and only 12% received durvalumab beyond progression [9].

In clinical practice, the two most important clinical factors influencing the choice of treatment after recurrence are the site of disease progression and the timing of relapse.

An exploratory analysis of the PACIFIC trial showed that durvalumab reduced the number of extra thoracic first progression. Approximately two out of three of patients had one or two distant lesions, most commonly in the brain [67]. These findings from PACIFIC trial are consistent with the results of several RW studies [55]. Patients with limited extra thoracic disease were therefore good candidates for locally ablative treatment, such as stereotactic radiotherapy. Based on the results of the phase II randomized trial SABR-COMET, the addition of stereotactic ablative radiotherapy to standard of care treatment improved OS without detrimental impact in quality of life in metastatic setting [68].

Thus, oligoprogression during durvalumab maintenance could be reasonably treated with local therapies while continuing immunotherapy. 

On the other hand, local treatment alone might not be enough in the case of oligo-recurrence/oligo-progression after the end of durvalumab treatment as the disease is likely to be spreading in the bloodstream as circulating tumor cells.

Salvage pulmonary resection of locoregional relapses represents a suitable option but only in a small and select fraction of patients and after multidisciplinary discussion [69].

For those patients progressing with full-blown metastatic disease in distant sites, there is not a defined standard systemic treatment, and, in daily clinical practice, the choice of systemic therapy is largely influenced by the concepts of platinum and immunotherapy resistance.

A retrospective study confirmed that in patients with locally advanced NSCLC relapsed after CRT, re-administration of a platinum-based doublet achieved worse PFS if relapse occurred within 6 months versus > 6 months after chemotherapy, respectively [70].

Likewise, disease progression occurring within 6 months of the last immunotherapy administration is the pivotal criterion in defining immunotherapy resistance [71]. 

The multicenter, retrospective analysis TOPGAN 2021-02 analyzed 127 patients who progressed after chemo-radiotherapy and durvalumab. Second-line treatments were categorized according to the time of progression. Surprisingly, in the early discontinuation group (patients with disease progression within 6 months after initiation of durvalumab), both platinum and non-platinum regimens were almost equally adopted, but patients with early relapse had a shorter PFS after platinum-based chemotherapy than those who relapsed after 6 months, confirming the concept of platinum resistance. Conversely, the rate of immune-checkpoint inhibitors-containing regimens as second-line treatment increased over time from early to later discontinuation of durvalumab (disease progression from 12 months after the initiation of durvalumab consolidation therapy) [50]. In other words, the longer the interval was after durvalumab discontinuation, the higher propensity was to use immunotherapy again.

In general, the maintenance or rechallenge of anti-PD1/PD-L1 or anti-CTLA-4 agents after the failure of first-line immunotherapy has gained very little clinical benefit [72], especially in patients who progressed during ICI treatment [73]. These findings constitute also the administrative constraints in keeping on with an immunotherapy-based strategy, at least in European countries.

According to most scientific guidelines, patients relapsing after chemo-radiation and durvalumab without any targetable gene alteration are, therefore, candidates for platinum-based chemotherapy (depending on the 6-month cutoff from last platinum dose) or other single-agent chemotherapies [74].

Second-generation immunotherapy trials with new agents modulating the tumor-microenvironment (TME), inducing activating immune checkpoints (DLL3, ICOS) or repressing inhibitory molecules different from PD1/PD-L1 or anti-CTLA-4 (LAG-3, TIGIT), enhancing MHC-mediated antigen presentation (STING), and activating different immune cell types (e.g., NK cells, Dendritic cells) all represent areas of research and hope for the future.

In the absence of prospective data, the best treatment option, at this time, seems to be the enrollment in clinical trials evaluating new treatment strategies stratified according to time of relapse.

## 4. Conclusions

Durvalumab has significantly reshaped the treatment landscape for stage III NSCLC, demonstrating substantial survival benefits in both clinical trials and real-world settings. This review highlights key prognostic factors, including PD-L1 expression, timely initiation post-CRT, and non-squamous histology, which influence patient outcomes. Despite these advances, challenges such as immune-related adverse events, treatment beyond progression, and the lack of optimal strategies for patients with driver mutations remain critical areas for improvement.

The heterogeneity of real-world patient populations underscores the need for more personalized treatment approaches. Additionally, the differences in study design and patient selection criteria complicate direct comparisons between clinical trials and real-world data. Variability in PD-L1 assessment methods, the timing of durvalumab initiation, and differences in concurrent treatments further contribute to these challenges.

Future research should focus on refining prognostic markers, integrating genomic profiling, and exploring novel therapeutic strategies, including biomarker-driven treatment selection. Prospective studies investigating second-line treatment options and strategies for managing disease progression post-durvalumab are essential to optimizing long-term patient outcomes. Ultimately, a multidisciplinary approach combining immunotherapy, advanced imaging techniques, and emerging biomarkers may pave the way for more effective and personalized treatment strategies in this challenging disease setting.

## Figures and Tables

**Table 1:** Patients’ characteristics in the analyzed studies.

	N° pts	Age, Median	Sex, Male (%)	Race	ECOG PS ≥ 2 (%)	Comorbidity	Smoking	Histology (%)
Guberina et al. [6]	39	62.3	67	Missing	8	COPD G ≥ 3 5%	Ever smoker 95%	SCC 43.6 Non-SQ 56.4
Moore et al. [7]	935	60	95	White 78% Black 21%	16	CCI median: 6 pt COPD: 70%	Current 46%	SCC 50 Non-SQ 43 Other 7
Waterhouse et al. [8]	528	70	51.5	White 70.6% Black 5.5% Missing 23.9%	9.1	Missing	Current 23.5% Ever smoker79.6%	SCC 45.3 Non-SQ 44.7 Other 10
Huang et al. [9]	39	64	79.5	97.4% Asiatic	5.1	Missing	Current 30.8% Ever smoker 79.5%	SCC 28.8 Non-SQ 61.1 Other 10.1
Preti et al. [10]	118	66,3	51.7	Missing	0.7	Missing	Current 24.7% Ever smoker 90.6%	SCC 32.2 Non-SQ 61.9 Other 5.9
Sankar et al. [11]	1006	69	Missing	White 74.1% African American 22% Other/unknown 3.9%	Missing	CCI G ≥ 6 51.3%	Current 43.2% Ever smoker 83.2%	SCC 48.2 Non-SQ 48.7 Other 3.1
Verschueren et al. [12]	106	64.2	50.9	Missing	0.9	Missing	Missing	SCC 30.8 Non-SQ 60.8 Other 8.4
Saad et al. [13]	71	67	63.7	Missing	5.6	Missing	Ever smoker 90.1%	SCC 32.4 Non-SQ 54.9 Other 12.7
Borghetti et al. [14]	85	69	75	Missing	0	COPD 57.1% COPD G ≥ 3 13.5%	Current 42.3% Ever smoker 92.3%	SCC 40.4 Non-SQ 55.8 Other 3.8
Avrillon et al. [15]	576	64	72.9	Missing	Missing	COPD 3.7%	Missing	SCC 39.9 Non-SQ 52.1 Other 8
Denault et al. [16]	134	66	59.7	Asian 8.2% Non Asian 91.8%	Missing	Missing	Missing	SCC 28.3 Non-SQ 70.1 Other 1.6
Girard et al. [17]	1399	66	67.5	Missing	2	Missing	Current 32.6% Ever smoker 92.1%	SCC 36 Non-SQ 64
Park et al. [18]	157	65	85.4	Missing	10.8	Comorbidity 72.6% COPD 31.8%	Current 32.5% Ever smoker 80.3%	SCC 52.2 Non-SQ 40.8 Other 7
Takeda et al. [19]	107	70	76	Missing	0	Missing	Missing	SCC 50 Non-SQ 43.9 Other 6.1
Gomez Rueda et al. [20]	244	67	79.9	Missing	3.6	COPD 23.8% Other Cancers 23.4%	Current 63.5% Former 33.2%	SCC 45.3 Non SQ 54.7
Stevens et al. [21]	152	67	63.2	Missing	4.6	COPD 32.9% Cardiovascular disease 56.6%	Current 67.7% Former 28%	SCC 32.2 Non SQ 57.2
Kakiuchi et al. [22]	208	69.5	80.3	Missing	1	Missing	Ever smoker 84.6%	SCC 40.9% Non-SQ 59.1
Trinh et al. [23]	79	63.5	43	Missing	Missing	Missing	Current 26.6% Former 68.4%	SCC 45.6% Non-SQ 48.1% Other 6.3%

**Table 2:** OS data for Durvalumab administration in the selected studies.

First Author	Year	Type of Article	Number of Patients	cCHT	sCHT	I Drug Carbo Platin	I Drug Cis Platin	II Drug Paclitaxel	II Drug Etoposide	II Drug Pemetrexed	II Drug Vinorelbina	Mono-CHT CBDCA	CHT 3-Week	CHT 1-Week	Dose < 54 Gy	Dose 54–66 Gy	Dose > 66 Gy	Dose 73.8 BIG	Dose 74 Gy	nFR	hFR	3D	IMRT	VMAT	IMRT SS	Median OS	1-Year OS	2-Years OS	3-Years OS
				%	%	%	%	%	%	%	%	%	%	%	%	%	%	%	%	%	%	%	%	%	%	months	%	%	%
Riudavets et al. [30]	2022	Retrospective	323	81	19	-	-	-	-	-	-	-	-	-	-	-	-	-	-	-	-	-	-	-	-	47	-	-	-
Gao et al. [31]	2022	Retrospective	190	87.3	12.6	83.7	15.3	80	13.2	4.7	0	0	-	-	-	93.7	5.3	-	-	-	-	-	-	-	-	-	87	-	60.3
Pennock et al. [32]	2023	Retrospective	59	100	0	100	0	100	0	0	0	0	0	100	11.8	-	-	-	-	68	32	-	-	-	-	32	-	-	-
Edwards et al. [33]	2023	Retrospective	1994	-	-	71	0	71	0	0	0	0	-	-	-	-	-	-	-	-	-	-	-	-	-	-	-	-	-
Wang et al. [34]	2022	Review	1677	83.7	16.4	-	-	-	-	-	-	-	-	-	-	-	-	-	-	-	-	-	-	-	-	-	90	-	-
LeClair et al. [35]	2021	Retrospective	83	-	-	78.4	17	70	9	14	0	0		-	-	85.5	-	-	-	-	-	-	-	-	-	-	-	-	-
Shirasawa et al. [36]	2021	Retrospective	551	-	-	-	-	-	-	-	-	13	-	-	-	100	5	-	-	-	-	-	-	-	-	51.8	-	-	-
Guo et al. [37]	2022	Retrospective	134	100	0	78.4	21.5	54	10.9	35	0	0	-	-	-	100	-	-	-	-	-	-	-	-	-	32.4	-	-	-
Takeda et al. [19]	2022	Retrospective	107	100	0	38.3	54.2	38.3	0	9.3	44.9	0	-	35.5	-	100	-	-	-	-	-	0	100	72.9	27.1	-	88	80	75
Abe et al. [38]	2023	Retrospective	29	100	0	-	-	-	-	-	-	-	-	-	-	100	-	-	-	-	-	66	-	34.5	0	-	84	-	-
Storm et al. [39]	2022	Retrospective	-	-	-	-	-	-	-	-	-	-	-	-	-	-	-	-	-	-	-	-	-	-	-	-	-	-	-
Girard et al. [17]	2022	Retrospective	1399	76.6	14.4	37.3	51.2	27.6	20.4	10.7	33.1	0.5	-	-	-	41.4	52.4	-	-	-	-	-	-	-	-	-	-	71.2	-
Park et al. [18]	2023	Retrospective	157	97.5	2.5	28	71.6	93	4.4	-	0.6	-	5.7	93	1.9	96.8	1.3	-	-	-	-	37	57.3	-	-	-	88	71	69.2
Li et al. [40]	2023	Review	4496	93.2	6.8	-	-	-	-	-	-	-	-	-	-	-	-	-	-	-	-	-	-	-	-	-	87	48.1	-
Yamamoto et al. [41]	2022	Retrospective	68	-	-	40	48	40	-	-	48	-	-	-	-	100	-	-	-	100	0	0	100	100	0	-	84	-	-
Taugner et al. [42]	2021	Prospective	33	51.5	33.3	-	82	-	-	-	82	-	-	-	-	100	-	-	-	100	0	0	100	100	0	-	87	-	-
Qiu et al. [43]	2023	Retrospective	353	-	-	-	-	-	-	-	-	-	-	-	-	-	-	-	-	-	-	-	-	-	-	-	-	-	-
Sankar et al. [11]	2022	Retrospective	1006	100	0	70.8	7.7	70.8	7.7	5.6	-	-	-	-	-	-	-	-	-	-	-	-	-	-	-	-	77	61.9	-
Wass et al. [29]	2022	Retrospective	78	46.2	53.8	70.6	29.4	0	0	52.9	29.5	3.5	-	-	-	46.2	-	53.8	-	-	-	0	100	100	0	-	-	-	-
Park, Jeon et al. [44]	2023	Retrospective	386	100	0	10.1	89.9	100	0	0	0	0	0	100	-	-	-	-	-	-	-	-	-	-	-	-	-	74.4	-
Moore et al. [7]	2023	Retrospective	935	98	2	88	11	-	-	-	-	-	-	-	3	78	8	-	1	-	-	-	-	-	-	-	-	-	-
Guberina et al. [6]	2022	Retrospective	160	96.9	3,1	5	78,1	-	-	-	81.2	-	-	-	0,6	-	5.6	-	-	-	-	0	100	-	-	-	-	80	-
Stana et al. [28]	2023	Retrospective	112	0	100	-	-	-	-	-	-	-	-	-	-	50	-	50	-	50	50	0	100	54	46	-	-	-	-
Preti et al. [10]	2023	Retrospective	118	100	0	50	44	33.8	27.11	-	0.8	-	-	-	-	-	-	-	-	-	-	-	-	-	-	-	-	-	-
Lebow et al. [45]	2023	Retrospective	81	100	0	69	29	38	7	53	0	0	-	-	-	-	-	-	-	100	-	-	-	-	-	-	93	72	-
Zhang et al. [24]	2023	Review	4056	92	8	-	-	-	-	-	-	-	-	-	-	-	-	-	-	-	-	-	-	-	-	-	85	-	-
Denault et al. [16]	2023	Retrospective	453	-	-	64.5	35.4	-	-	-	-	-	-	-	-	97.6	-	-	-	-	-	-	-	-	-	37.9	-	71.5	-
Spigel et al. [46]	2022	Prospective	713	100	0	42.2	55.4	-	-	-	-	-	-	-	-	-	-	-	-	-	-	-	-	-	-	47.5	83	66.3	56.7
Borghetti, Volpi et al. [14]	2023	Retrospective	85	87.1	12.9	82.3	3.5	82.3	3.5	-	-	-	52	48.2	-	100	-	-	-	100	-	-	100	94.1	5.9	52	83	69.4	-
Liu, Zhang et al. [47]	2022	Retrospective	104	100	0	91	9	85	3	10	0	1	-	-	-	85	12	-	-	-	-	-	-	-	-	36.2		-	-
Bryant et al. [25]	2022	Retrospective	1306	100	0	71.6	8	-	-	2.9	-	-	-	-	-	-	-	-	-	-	-	-	-	-	-	-	-	-	-
Liu, Bratton et al. [48]	2022	Retrospective	5802	100	0	-	-	-	-	-	-	-	-	-	-	-	-	-	-	-	-	-	-	-	-	-	-	-	-
Diamond et al. [49]	2023	Retrospective	62	100	0	61	-	61	-	-	-	-	-	-	-	100	-	-	-	-	-	0	100	-	-	-	87	-	-
Hasegawa et al. [50]	2023	Retrospective	127	98.4	1.6	41.7	41	41.7	-	-	13.4	-	-	-	-	-	-	-	-	-	-	-	-	-	-	18.2	-	-	-
Huang et al. [9]	2022	Retrospective	84	100	0	28,6	63.1	26.2	38.1	27.4	-	-	-	-	-	100	-	-	-	100	0	0	100	-	-	-	94	75.2	-
Yang et al. [51]	2023	Review	2560	-	-	-	-	-	-	-	-	-	-	-	-	100	-	-	-	-	-	-	-	-	-	-	-	-	-
Waterhouse et al. [8]	2023	Retrospective	528	100	0	86.6	10.2	86.6	10.2	-	-	-	-	-	3,2	80.3	-	-	-	-	-	-	-	-	-	-	84	64	-
Verschueren et al. [12]	2023	Retrospective	383	69.7	30.3	-	-	-	-	-	-	-	-	-	-	-	-	-	-	-	-	-	-	-	-	-	84	-	-
Saad et al. [13]	2022	Retrospective	215	96	4	67.4	11.6	67.4	11.6	-	-	-	-	-	-	100	-	-	-	-	-	49	51.2	-	-	-	86	-	-
Avrillon et al. [15]	2022	Retrospective	576	100	0	42.9	52.9	27.9	2.1	15.9	45	-	-	-	-	-	-	-	-	-	-	-	-	-	-	-	-	-	-
Borghetti, Imbrescia et al. [52]	2022	Retrospective	24	75	25	66.7	33	37.5	12.5	12.5	16.7	0	58	41.7	-	-	-	-	-	-	-	-	-	-	-	-	-	-	-
Boys et al. [53]	2022	Retrospective	126	100	0	67	30	67	29	0.08	-	-	-	-	10	90	-	-	-	100	0	0	100	-	-	58.7	-	-	-

cCHT—concurrent CHT; sCHT—sequential CHT; IMRT—Intensity-Modulated Radiation Therapy; VMAT—Volumetric Modulated Arc Therapy; IMRT SS—Intensity-Modulated Radiation Therapy Step and Shoot; nFR—Normo fraction (30 fractions − 2 Gy/fraction); hFR-Hypofraction (20–25 fractions); - not reached.

**Table 3:** Data about pneumonitis in the selected studies.

	RT Technique	RT Dose (Median)	RT Dose Range	Lung V20 Median (%)	Lung V20, <30% (%)	Lung V20 ≥30% (%)	MLD Median (Gy)	MLD ≤18 Gy	MLD	MHD (Median)	MHD <10 Gy	MHD ≥10 Gy	N. of Cycles of Durvalumab (Median)	N. of Cycles of Durvalumab (Range)	Time to Durvalumab (Median, Days)	Time to Durvalumab (Range, Days)	Pneumonitis	G2+ Pneumonitis	G2+ Pneumonitis RT	G2+ Pneumonitis DURVA	G2+ Pneumonitis EQUIVOCAL	Grade 3+ Pneumonitis	Discontinued Durva Due to Pneumonitis
Diamond et al. [49]	-	-	60–64.8	27	56%	44	15.5	77	23	9.50 Gy (6.25–14)	55	45	17	8–24	-	-	42	32.30	24	6.50	1.6	9.7	11
Avrillon et al. [15]	-	-	45–74	-	-	-	-	-	-	-	-	-	16	1–37	36	0–157	-	-	-	-	-	-	-
Huang et al. [9]	VMAT/IMRT	-	60–66	-	-	-	-	-	-	-	-	-	13	-	38		28.2	-	-	-	-	7.7	-
Waterhouse et al. [8]	-	-	54–66	-	-	-	-	-	-	-	-	-	-	-	47	0–434	-	-	-	13.4	-	-	-
Saad et al. [13]	VMAT/IMRT	-	56–66	-	-	-	-	-	-	-	-	-	-	-	-	-	49.3	-	-	-	-	5.6	-
Guberina et al. [6]	VMAT/IMRT	-	53.5–74	-	-	-	15.2	-	-	-	-	-	18	1–27	25	7–80	-	-	-	-	-	-	10.25
Borghetti et al. [14]	VMAT/IMRT	60	-	-	-	-	-	-	-	-	-	-	-	-	47	2–105	30.8	23.1	-	-	-	5.8	3.85
Preti et al. [10]	-	-	-	-	-	-	-	-	-	-	-	-	-	-	40.5	7–238	39.8	38.9	0	38.9	0	16.9	-
Stana et al. [28]	VMAT/IMRT	72.3	39–88.2	20	-	-	12	-	-	-	-	-					15.6	15.6			-	2	
Park et al. [18]	-	-	54–66	-	-	-	-	-	-	-	-	-	-	-	-	-	-	-	85.7	-	-	-	-
Wass et al (high dose arm) [29,43]	VMAT	72	-	20.5	-	-	13	-	-	-	-	-	14	1–26	18.5	4–127	28.6	-	-	-	-	-	-
Wass et al (standard of care arm) [29]	VMAT	59,4	-	16	-	-	16.7	-	-	-	-	-	8	1–21	22	2–114	27.8	-	-	-	-	-	-
Taugner et al. [42]	-	63,6	-	-	-	-	-	-	-	-	-	-	14	2–24	25	13–103	-	-	-	-	-	15.38	-
Abe et al. [38]	VMAT/3D	60	-	21.5	-	-	12.4	-	-	11.7	-	-	7	-	-	-	100	50	-	-	-	0	25
Park et al. [44]	VMAT/3D/IMRT	-	54–66	-	-	-	-	-	-	-	-	-	19	1–32	32	0–86	36.6	-	36.3	14.6	-	1.9	-
LeClair et al. [35]	-	60	-		-	-		-	-	-	-	-	13.9	1–47	57.3	8–226	25.3	22.9	2.4		-	6	22.9
Edwards et al. [33]	-	-	-	-	-	-	-	-	-	-	-	-	-	-	-	-	20.9	-	5.57	9	6.27	-	-
Gao et al. [31]	IMRT/IMPT	60	-		-	-		-	-	-	-	-	12	4–22	35.5	27–52		26.3			-		7.4
Riudavets et al. [30]	-	-	-	-	-	-	-	-	-	-	-	-	-	-	-	-	-	-	-	21	-	4.6	-
Miura et al. [57]	-	60	54–60	18.9	-	-	-	-	-	-	-	-	-	-	11	1–42	61	58.5	-	-	-	2.4	53.7
Inoue et al. [58]	3D/IMRT/PRB	-	60–64	22	-	-	-	-	-	-	-	-	-	-	-	-	73.3	46.7	-	-	-	0	-
Saito et al. [59]	3D	-	-	-	-	-	-	-	-	-	-	-	-	6.5	11	1–39	-	-	-	-	-	-	47.2
Jung et al. [60]	3D/IMRT/PRB	-	-	-	-	-	-	-	-	-	-	-	-	-	-	-	81	42.9	-	-	-	14.3	42.9
Mayahara et al. [61]	3D/IMRT	60	54–60	-	-	-	-	-	-	-	-	-	-	-	-	-	89.3	39.3	-	-	-	8.9	39.3

## Data Availability

Research data are stored in an institutional repository and will be shared upon request to the corresponding author.
